# Integrating a Multimode Design Into a National Random-Digit–Dialed Telephone Survey

**Published:** 2011-10-15

**Authors:** Shaohua Sean Hu, Carol Pierannunzi, Lina Balluz

**Affiliations:** Office on Smoking and Health, National Center for Chronic Disease Prevention and Health Promotion, Centers for Disease Control and Prevention; Division of Behavioral Surveillance, Centers for Disease Control and Prevention, Atlanta, Georgia; Division of Behavioral Surveillance, Centers for Disease Control and Prevention, Atlanta, Georgia

## Abstract

The Behavioral Risk Factor Surveillance System (BRFSS) was originally conducted by using a landline telephone survey mode of data collection. To meet challenges of random-digit–dial (RDD) surveys and to ensure data quality and validity, BRFSS is integrating multiple modes of data collection to enhance validity. The survey of adults who use only cellular telephones is now conducted in parallel with ongoing, monthly landline telephone BRFSS data collection, and a mail follow-up survey is being implemented to increase response rates and to assess nonresponse bias. A pilot study in which respondents' physical measurements are taken is being conducted to assess the feasibility of collecting these data for a subsample of adults in 2 states. Physical measures would allow for the adjustment of key self-reported risk factor and health condition estimates and improve the accuracy and usefulness of BRFSS data. This article provides an overview of these new modes of data collection.

## Introduction

The Behavioral Risk Factor Surveillance System (BRFSS) is a state-based random-digit–dial (RDD) telephone surveillance system that was established in 1984 by the Centers for Disease Control and Prevention (CDC) and state health departments. Information regarding health risk behaviors, clinical preventive health practices, and health care access, primarily related to chronic disease and injury, is obtained from a representative sample of adults in each state. For most states, BRFSS is the only source for this type of information. Data are collected monthly in all 50 states, the District of Columbia, Puerto Rico, the Virgin Islands, and Guam. Approximately 400,000 adult interviews are completed each year, making BRFSS the largest health telephone survey conducted in the world (1). Not only is BRFSS a unique source of risk behavior data for states, but it is also useful in measuring progress toward *Healthy People 2020* objectives for the states and the nation (2).

For more than 30 years, RDD landline telephone surveys have been the workhorse of the survey research industry. During the past decade, however, participation in most RDD telephone surveys has declined because of changes in personal communication technologies, growth of call-screening technologies, and heightened privacy concerns resulting from increased number of telemarketing calls (3,4). Additionally, coverage provided by landline RDD survey samples has increasingly been questioned. RDD landline frames exclude households that do not have a telephone of any type (approximately 2% in 2009) (5). The increased use of cellular telephones has exacerbated this problem; 24.5% of households were reported to be cellular telephone–only (ie, households with no landline telephone) during the second half of 2009 (5-9).

As an RDD landline telephone survey, BRFSS has several specific challenges. First, households with only cellular telephone coverage or that lack landline telephones were not included; therefore, BRFSS may have been excluding people of low socioeconomic status. Second, the survey response rates have been declining over the past several years, making it increasingly difficult to collect survey data by using RDD landline telephone methods. Third, data are self-reported and are subject to recall bias.

RDD surveys are typically conducted using only the telephone survey mode of data collection. However, to meet challenges of increasing nonresponse and noncoverage rates because of households that use only cellular telephones and to facilitate validation of key BRFSS interview questions, beginning in 2009, BRFSS gradually integrated a multimode design into its RDD landline telephone survey. A survey of adults who use only cellular telephones was conducted in parallel with the ongoing landline-based health survey to reduce noncoverage and related bias in key estimates. A mail follow-up survey to nonrespondents of the RDD landline telephone survey was implemented in 6 states in 2010 to raise response rates and to assess nonresponse bias. A physical measurement study was piloted in 2 states in 2010 to adjust for recall bias with self-reported data. We describe changes that have been made as well as changes that are being implemented in BRFSS operations and comment on the effect these changes have had on coverage, response rates, and related survey bias.

## BRFSS Multimode Designs

Multimode survey designs have been in use for a long time and have become standard in some countries, as survey managers seek to use collection procedures that produce the best possible data in the constraints of time and budgets (10). Multimode surveys combine different modes of data collection, including in-person, telephone, Internet, and mail.

Cross-sectional surveys use 3 primary types of multimode survey design (10,11). In the first, often referred to as *mode assignment*, a sample can be divided into subsets defined in some manner, and different modes can be applied to the subsets. For example, the population of adults living in households with telephone service can be divided into 2 primary strata: landline and cellular phone–only (12). A second multimode design is referred to as *sequential*. Potential respondents who do not respond to the first mode are contacted to respond via a second mode. Mail surveys used following telephone contact are examples of sequential design. The third type of multimode design is a *concurrent* mode approach, which involves offering the respondent multiple channels at once for completing the survey (eg, via telephone, using the Internet). The respondent then chooses the preferred mode of responding. An example of this type of design would be to simultaneously offer potential respondents the ability to respond via the Web or the telephone.

BRFSS assessed the potential costs and benefits in deciding to use a multimode design and is using pilot studies to determine the best protocols for each of the modes adopted. For the BRFSS pilot studies, new modes are tested in a limited number of states before widespread adoption and use.

### Cellular telephone surveys

For the past several decades, RDD telephone sampling of households with landline telephones provided a cost-efficient strategy for conducting surveys of the US population. However, as the percentage of cellular telephone–only households continues to grow, validity of the RDD landline sampling model used by most survey organizations has been questioned (6-9,13-15). The percentage of US adults who live in cellular telephone–only households increased by more than 700% between early 2003 (2.9%) and late 2009 (24.5%) (5). Specific subpopulations, such as renters, male respondents, minorities, and people living at or near the federal poverty level, are more likely to live in cellular telephone–only households, and wireless substitution is particularly high among young adults (people aged 18-34 y) (5,16-18). These adults are not covered by current RDD landline sampling procedures, which exclude telephone exchanges used for cellular telephones. As the percentage of cellular telephone–only households continues to grow, undercoverage bias poses a serious threat to the validity of landline RDD telephone surveys. In 2008, to address the trend of increasing prevalence of cellular telephone–only households and the corresponding potential for bias in estimates from surveys that sample only from landline frames, BRFSS initiated a pilot study in 18 states that used a survey of both landline and cellular telephone numbers. This study assessed the feasibility of conducting surveys using sampled cellular telephone numbers and expanded the research on the similarities and differences between respondents interviewed by landline versus cellular telephones.

The survey of adults who use only cellular telephones was conducted in parallel with ongoing, monthly landline BRFSS data collection. The sample was selected by screening a larger sample of cellular telephone numbers because adults who use only cellular telephones cannot be identified in advance of sampling. The BRFSS pilot studies found that outcome measures were significantly biased for 9 of 16 key health indicators resulting from exclusion of adults with only cellular telephones from the landline telephone–based survey (12). As landline telephone noncoverage rates for adults who use only cellular telephones continue to increase, these biases are likely to increase proportionally. Sampling and interviewing adults who use only cellular telephones are now a necessity if surveys by telephone are to provide valid, reliable, and representative data. Beginning in 2009, BRFSS expanded its traditional landline telephone–based RDD survey to a dual frame survey of landline and cellular telephone numbers in all 50 states.

### Mail follow-up survey

As part of efforts to explore alternative data collection methods for BRFSS and building on results from the BRFSS Mail Survey Pilot conducted in 2005 and 2006, the Mail Follow-up Survey (MFS) was designed and implemented in 2010. The MFS was designed to assess the effect of multimode data collection — specifically, an RDD landline telephone survey with mail survey follow-up of nonrespondents — on BRFSS response rates.

The primary goal of the MFS is to increase overall participation in BRFSS, especially among underrepresented groups, including young adult, male, minority, and working populations (16,17). For nonrespondents, the first step is to do a reverse match to obtain residential addresses for sample telephone numbers. On the basis of using commercial address databases for reverse matching and according to BRFSS experience, matches can be obtained for 50% to 70% of sample telephone numbers. A mailed questionnaire package is sent to the addresses of nonrespondents with an address match. For telephone numbers without an address match and where the adult respondent has been selected and the interview has not been completed (excluding partial interviews), the mailed questionnaire package is sent to the address resulting from information obtained from the sample household after states finish maximum calling attempts on the sample telephone number.

This approach uses the mailed survey as a nonrespondent follow-up technique. It offers the advantage of obtaining the full BRFSS interview (core and modules) for all of the telephone respondents before implementation of the MFS protocols. Previous findings indicated that the use of sequential multimodes makes the reporting task easier for respondents, which leads to higher response rates and better quality data (10). In 2011, more than a dozen states are implementing the MFS.

### Physical measurement pilot study

To collect information about risk factors and health conditions, BRFSS relies primarily on respondents' self-reported data, which are subject to various types of reporting error. The primary objective of the physical health measurements pilot study is to assess the feasibility of introducing actual measurements for a subsample of adults in each state to adjust key self-reported risk factor and health condition estimates and improve the accuracy and usefulness of BRFSS data.

A collaborative effort between the 50 states and CDC, the BRFSS surveys are a valuable source of information on health risk behaviors. Adding physical measures to the behavioral measures collected by telephone surveys will improve the reliability and validity of BRFSS data, enhance these data for use by state and federal health programs, and provide survey participants with more direct health information.

The concept of adjustment through the use of "verification" information can be accomplished with the use of a 2-phase sample. In 2-phase sampling, the verification information (ie, the physical health measurements from the phase 2 subsample), which is more costly to collect, is obtained only for a subsample of the full sample (19). The verification information can be used to adjust the self-reported data (from the phase 1 sample) at the aggregate level by using estimators found in the classic sampling texts by Kish and Cochran (20,21). If the correlation between the self-reported data collected during phase 1 and the physical measurement data collected during phase 2 for the subsample is high, then the sample size of physical health measurements can be small relative to the sample size of self-reports. This is important because the unit cost of obtaining 1 physical health measurement is almost always considerably higher than the cost of obtaining 1 self-report.

BRFSS collected physical health measurements for a subsample of adults in 2 pilot states during 2009 and 2010. To collect data, trained health professionals are sent to peoples' homes (or place of their choosing) to obtain health measurements (eg, height and weight, blood pressure, total cholesterol, fasting plasma glucose), which are reflective of the content of the 2009 BRFSS questionnaire. An incentive of $50 per respondent was provided.

The self-reported data that are collected during the first phase sample are less expensive and less accurate measures than are the physical measurements that are obtained from respondents during the second phase sample. A regression estimate can take advantage of both of these measures to produce more accurate overall estimates than could be obtained by either alone.

## Discussion

BRFSS is a valuable system for public health, and maintaining and ensuring its high quality is a priority for CDC and state health departments. However, BRFSS is facing many challenges and, to meet them, has expanded a traditional RDD landline telephone–based survey approach to one that uses multiple modes of data collection.

Using the multimode design reduces noncoverage rates and does lead to higher response and better-quality data, but compared with a single-mode design, it poses several problems. A sequential approach could substantially lengthen the field period. The use of multiple modes may raise issues of comparability across modes. For example, questions asked by an interviewer over the telephone, as opposed to being asked on paper, may be more likely to invoke socially desirable responses (22). Furthermore, questions asked on paper are more likely to ensure privacy and allow the respondent to complete the survey at his or her convenience. However, complex forms in which some questions are to be skipped cannot be used, and literacy issues must be considered regarding the MFS. Finally, landline and cellular telephone modes are used for various subsets of the sample, making it difficult to determine whether there is a mode effect. Evidence exists that survey mode can affect respondents' answers to questions, even when questions are worded identically (23). Attention should be paid to mode effects when analyzing data.

BRFSS currently includes cellular telephone frames in all states. The cellular phone survey does help BRFSS reach a higher percentage of underrepresented groups including young adult, male, minority, and working populations. However, the sample size is small (<10% of all completed surveys per state) because of higher costs associated with cellular telephone interviews. The sample size for the cellular telephone should be increased to match Cochran's estimate of optimal cellular telephone proportion of the sample (24,25) when more funding is available. This increase would set the proportion of completed interviews for cellular telephone respondents at 10% to 20% for each state. Furthermore, concern for the burden on the respondent in completing a 10- to 15-minute cellular telephone interview has restricted cellular telephone interviews in some states to the key component of the BRFSS questionnaire (core questions). In some piloted areas, the addition of 3 to 4 minutes of module questions has not had an effect on response rates among cellular telephone users. Therefore, research will continue on the optimal length of cellular telephone interviews by testing the addition of 3- to 4-minute modules, incrementally.

BRFSS is a powerful tool for building health-promotion activities, and the system is a critical part of public health in the United States. In the future, an additional mode of data collection will be introduced. Respondents will be provided the opportunity to respond via Web-based versions of BRFSS. BRFSS will pilot Web-based applications in 2011 and 2012 and assess the viability of online modes of data collection. BRFSS staff members at CDC and state levels will continue to address these issues to improve the utility of BRFSS data. The use of multiple modes of data collection is an increasing trend that offers BRFSS the possibility of compensating for critical issues faced by traditional RDD landline telephone surveys — either to reduce coverage error or to address other challenges that now affect BRFSS, such as increasing nonresponse rates and biases inherent in self-reported data.
